# Whole-body dynamic FDG-PET/CT parametric imaging in alveolar echinococcosis

**DOI:** 10.1007/s12149-025-02113-9

**Published:** 2025-10-06

**Authors:** Lars Husmann, Bert-Ram Sah, Fotis Kotasidis, Alexander Maurer, Cordula Meyer zu Schwabedissen, Ansgar Deibel, Martin W. Huellner

**Affiliations:** 1 Department of Nuclear Medicine, University Hospital Zurich, University of Zurich, Raemistrasse 100, CH-8091 Zurich, Switzerland; 2 Department of Diagnostic, Interventional, and Pediatric Radiology, Inselspital, University of Bern, Bern, Switzerland; 3 GE Healthcare, Waukesha, WI USA; 4 Division of Gastroenterology and Hepatology, University Hospital Zurich, University of Zurich, Zurich, Switzerland

**Keywords:** PET/CT, FDG, Dynamic PET, Patlak analysis, Parametric imaging, Alveolar echinococcosis

## Abstract

**Objective:**

To determine the role of whole-body dynamic (WBD)/Patlak parametric ^18^F-fluorodeoxyglucose (FDG) positron emission tomography/computed tomography (PET/CT) in patients with alveolar echinococcosis (AE). This technique allows separating metabolized from unmetabolized FDG in the blood pool and tissue, potentially providing complementary qualitative information and superior quantification to standard static PET/CT images.

**Methods:**

We prospectively analyzed 20 PET/CT datasets performed for staging or therapy monitoring in patients with confirmed AE. Dynamic and standard static PET/CT datasets were acquired in all patients, and quantitative imaging parameters were measured in the lesion with the highest uptake (i.e., maximum standardized uptake value (SUVmax) and Patlak parameters) and compared to normal liver tissue (SUVratio and Patlak ratio).

**Results:**

Mean SUVmax in AE manifestations was 5.7 ± 3.1 (3.2–13.9), compared to 3.2 ± 0.4 (2.5–4.2) in non-infected liver tissue, respective values for Patlak were 13.0 ± 8.6 (2.7–35.5) and 4.9 ± 2.8 (0.6–12.1). SUVratio (1.8 ± 1.1; 1.0–5.2) was significantly lower (*P* < 0.001) than Patlak ratio (3.2 ± 3.2; 1.1–15.6). Both ratios correlated significantly with *E. granulosus* hydatid fluid (EgHF) antibodies (SUVratio *r* = 0.73, *P* < 0.001; Patlak ratio *r* = 0.85, *P* < 0.001).

**Conclusion:**

WBD PET/CT yields higher lesion-to-background contrast and may, therefore, have the potential to increase sensitivity in the assessment of hepatic AE.

## Introduction

Alveolar echinococcosis (AE) is a rare zoonotic parasitosis [1] that typically manifests as a slow-growing tumor-like liver lesion. Advances in treatment have significantly improved patient prognosis. Complete surgical resection offers a potential cure [2, 3], while in advanced or inoperable cases, benzimidazole therapy has been shown to effectively control the disease and substantially enhance patient outcomes [4–9]. However, certain aspects of AE management remain without expert consensus or strong evidence, including: i) the optimal imaging modality for disease staging, ii) the management of patients experiencing significant morbidity due to benzimidazole therapy side effects, iii) the discontinuation of “lifelong” benzimidazole therapy, as evidence suggests that treatment may have parasitocidal effects, allowing for safe termination in select cases [10, 11].

A main challenge in AE management includes the inability to directly assess parasite vitality. Instead it is indirectly derived from parameters quantifying the host’s immune response, including anti-Em18 serology and perilesional metabolic activity on ^18^F-fluorodeoxyglucose (FDG) positron emission tomography/computed tomography (PET/CT) imaging [10, 11]. These parameters, however, suffer from certain limitations. Anti-Em18 serology in the real-world setting and perilesional metabolic activity determined by FDG-PET/CT suffer from low sensitivity, the latter demonstrated by variable measurement outcomes over time and more frequent detection of residual activity in delayed imaging [12, 13]. Treated AE lesions typically show only faint FDG uptake, which is further obscured by the relatively high liver background, making their assessment challenging with standard static PET/CT.

A recently introduced PET/CT imaging technique, known as whole-body dynamic (WBD) PET/CT (or Patlak parametric imaging), enables differentiation between metabolized FDG and unmetabolized FDG in the blood pool and tissue [14, 15]. This technique may enhance lesion-to-background contrast, potentially improving the sensitivity and accuracy of PET/CT for staging and therapy monitoring in hepatic AE [16].

Thus, our study aimed to evaluate the role of WBD/Patlak parametric PET/CT in patients with AE.

## Methods

### Study design and data collection

This prospective study analyzed 20 PET/CT scans from patients with confirmed AE, who were referred for staging or therapy monitoring between 2022 and 2024 at the ***anonymized for review***. Relevant clinical data, including patient demographics, laboratory results, and clinical and treatment information, were collected at the time of PET/CT.

The study protocol was approved by the local ethics committee (BASEC-Nr. 2022-01493) and all patients provided written informed consent. All procedures were conducted in accordance with the 1964 Helsinki declaration and its later amendments or comparable ethical standards.

### Imaging data acquisition

Patients fasted for at least 4 h, and body weight, height, and blood glucose level were measured prior to the examination. FDG dosage was body-weight adjusted and injected on the scanner bed (6-ring Discovery MI Gen2, GE Healthcare). Dynamic and standard static PET/CT data sets were acquired in all patients. Dynamic imaging started either directly after injection, with a single bed covering the heart being acquired for 10 min (12 × 5 s, 4 × 10 s, 8 × 25 s, 5 × 60 s), followed by 11 whole-body dynamic frames over 46 min (5 beds, 50 s/bed), or following an initial uptake time of 26 min followed by 7 frames over 29 min. Following the end of the dynamic acquisition, a static acquisition at 60 min post-injection (1.5 min/bed) was then performed. Reconstructed images, along with an aortic input function (IF), were used to generate MR_FDG_ images (DynamicIQ; GEHealthCare), based on traditional Patlak analysis (11 frames, full IF) and relative Patlak analysis (7 frames, partial IF). The input function was corrected for blood to plasma partition and fitted with a bi-exponential model while preserving the area under the curve for the first 10 min where continuous sampling was available in standard Patlak analysis. Standard static PET/CT imaging was performed at 60 min post-injection, without changing the position of the patient in the arms-down position. A non-enhanced CT scan was used for attenuation correction.

### Data analysis and definitions

Quantitative imaging parameters were measured in the lesions exhibiting the highest FDG uptake (i.e., maximum standardized uptake value (SUVmax) as well as metabolic rate of FDG (MR_FDG_)), and compared to normal/non-infected liver tissue (SUVratio and Patlak ratio). In line with a commonly used approach for assessing hepatic background—such as in lymphoma evaluation—we utilized SUVmax as the reference parameter [17]. Measurements were performed by two experienced, doubly board certified nuclear medicine physicians and radiologists using a dedicated workstation (Advantage Workstation, Version 4.7; GE HealthCare Biosciences, Pittsburgh, PA). In case of discrepancy, the data was reanalyzed by both readers, and a consensus was reached.

In addition, the readers quantified the number of detectable hepatic and extrahepatic lesions, as well as the size of the largest liver lesion. Disease extent was staged according to the PNM classification (P = parasitic mass in the liver; N = involvement of neighboring organs; M = metastasis) as defined by the WHO Informal Working Group on Echinococcosis [2, 18–20]. The presence of calcifications within AE manifestations was categorized as none, mild, intermediate, or extensive.

Finally, serum samples, taken at the time of the PET/CT, were analyzed at the Institute of Parasitology, University of Zurich, using Em-18 antigens. Enzyme-linked immunosorbent assays (ELISA) for *E. granulosus* hydatid fluid (EgHF) were carried out as previously described by Kronenberg et. al [21].

### Statistical analyses

Statistical analyses were conducted using commercially available software (IBM SPSS Statistics, Version 30). Quantitative variables were reported as mean ± standard deviation (range), while categorical variables were reported as frequencies and percentages. Differences in SUVratio and MR_FDG_ ratio in AE manifestations were tested for significance using Mann–Whitney U test. Pearson correlation coefficients were calculated to compare absolute values of SUVratio and MR_FDG_ ratio with Em-18 and EgHF antibodies. A *P* value of ≤ 0.05 was considered to indicate statistical significance.

## Results

### Patient population

PET/CT was performed in 20 patients with serology-confirmed alveolar echinococcosis for staging and assessment of metabolic activity at baseline (*n* = 7) or restaging or as part of a structured treatment discontinuation (*n* = 13). The mean age of the patients was 52 ± 17 years (range: 22–80), with a mean weight of 70 ± 12 kg (range: 52–95) and a mean height of 172 ± 7 cm (range: 160–188). Sixty percent (*n* = 12) of the patients were female. None of the patients had elevated blood glucose levels prior to the PET/CT examination (mean4.9 mmol/L (range: 4.7–6.7). A mean dose of 174 ± 61 MBq FDG (range: 103–302) was administered. The demographics of the patient population are given in Table 1.

**Table 1:** Patient demographics of all patients

ID	Age	Gender	Weight in kg	Height in cm	PNM	Days between initial diagnosis and PET/CT	Number of lesions	Size of biggest lesion (mm)	Calcifications	Em-18 in AU	EgHF in AU	SUVmax	Patlak	SUV ratio	Patlak ratio
01	36	Female	61	167	P1N0M0	61	5	42	Mild	0	27	3.2	5.3	1.0	1.1
02	80	Male	89	177	P4N1M1	21	1	125	Intermediate	42	31	9.4	18.5	2.9	4.0
03	56	Female	58	169	P2N0M0	121	2	32	Mild	0	0	3.4	11.4	1.1	1.7
04	43	Female	58	165	P4N0M0	1181	7	37	Extensive	0	3	3.2	12.8	1.0	1.1
05	71	Male	76	172	P4N0M0	3963	1	95	Extensive	1	16	5.2	11.7	1.5	2.7
06	55	Female	65	170	P4N1M0	5034	3	92	Extensive	42	59	11.2	43.3	2.7	4.3
07	77	Female	54	167	P4N1M0	2087	> 20	56	Extensive	1	41	3.5	22.3	1.1	2.3
08	75	Female	60	160	P4N0M0	3240	2	102	Extensive	0	12	5.3	18.8	1.5	1.5
09	60	Male	90	188	P4N1M0	4004	2	61	Intermediate	18	27	7.3	28.8	2.5	4.2
10	26	Female	52	164	P2N0M0	10	1	58	Intermediate	5	28	4.1	15.7	1.3	2.6
11	40	Female	78	176	P4N1M0	2318	1	105	Intermediate	0	25	4.2	5.9	1.3	1.9
12	35	Male	70	186	P2N0M0	851	1	95	Extensive	0	20	3.5	8.3	1.1	2.1
13	39	Female	75	166	P4N0M0	3110	1	53	Extensive	1	8	3.9	5.2	1.0	2.0
14	22	Female	65	168	P2N1M0	255	1	129	Extensive	n.a	n.a	9.1	2.7	2.5	4.6
15	58	Male	86	182	P2N1M0	2318	2	58	Intermediate	4	11	3.9	6.1	1.3	2.0
16	65	Male	69	173	P2N1M0	41	2	85	Mild	108	19	8.6	18.0	3.4	5.2
17	71	Male	62	178	P2N0M0	1443	2	57	Intermediate	0	10	4.0	6.2	1.3	1.6
18	49	Male	70	168	P3N0M0	29	1	95	Intermediate	0	106	13.9	22.2	5.2	15.6
19	54	Female	95	175	P1N0M0	55	1	67	Extensive	2	46	4.2	11.2	1.2	2.8
20	35	Female	59	174	P4N0M0	1911	2	16	Mild	0	1	3.2	4.8	1.1	1.6

*AU* Antibody units, *CT* Computed tomography, *EgHF* E. *granulosus* hydatid fluid, *Em-18* levels of Em-18 antibodies, *FDG* ^18^F-fluorodeoxyglucose, *ID* patient identification, *n.a.* not applicable, *PNM* (PNM stage *P* = parasitic mass in the liver, *N* = involvement of neighboring organs, *M* = metastasis), *SUVmax* maximum standardized uptake value

At the time of the PET/CT examination, 16 patients (80%) were undergoing benzimidazole therapy. In three of the patients not on benzimidazole therapy, treatment was initiated after the PET/CT, while in one patient, treatment was stopped just prior to the PET/CT and has not been reinitiated since.

#### PET/CT

**Fig. 1:**
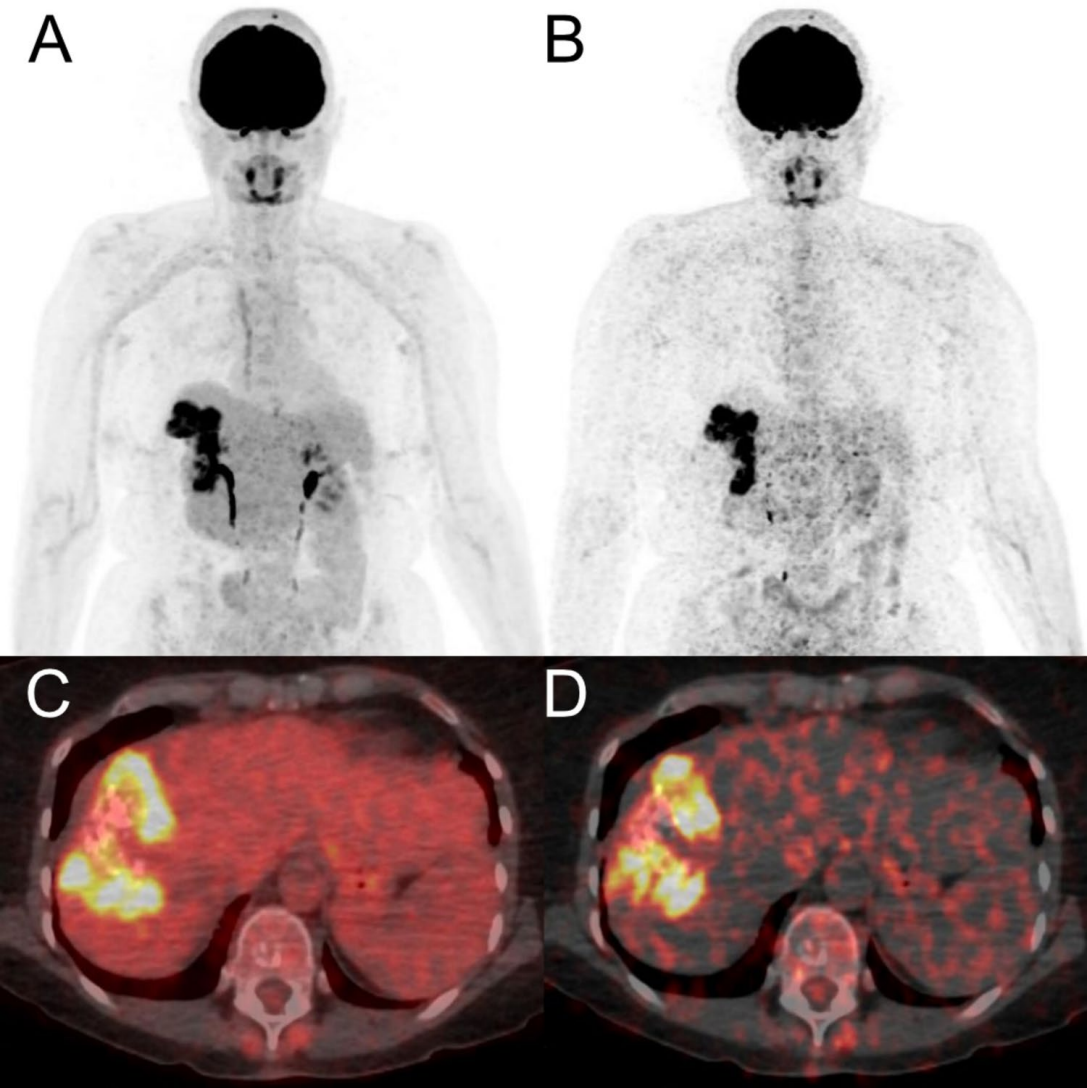
PET/CT performed for therapy monitoring of AE in a 55-year old woman (patient 06 in Table 1) showed intense metabolic activity (in maximum intensity reconstructions of PET, **A** and **B**) and fused PET/CT images (**C** and **D**) in a partially calcified lesion in the right liver lobe. In standard static PET/CT images (A and C), SUVmax of the AE manifestation was 11.2, and the lesion-to-background ratio (SUVratio) was 2.7. For WBD PET/CT (**B** and **D**), the respective value in the AE manifestation was 43.3, and the lesion-to-background ratio was comparatively higher (Patlak ratio 4.3)

**Fig. 2:**
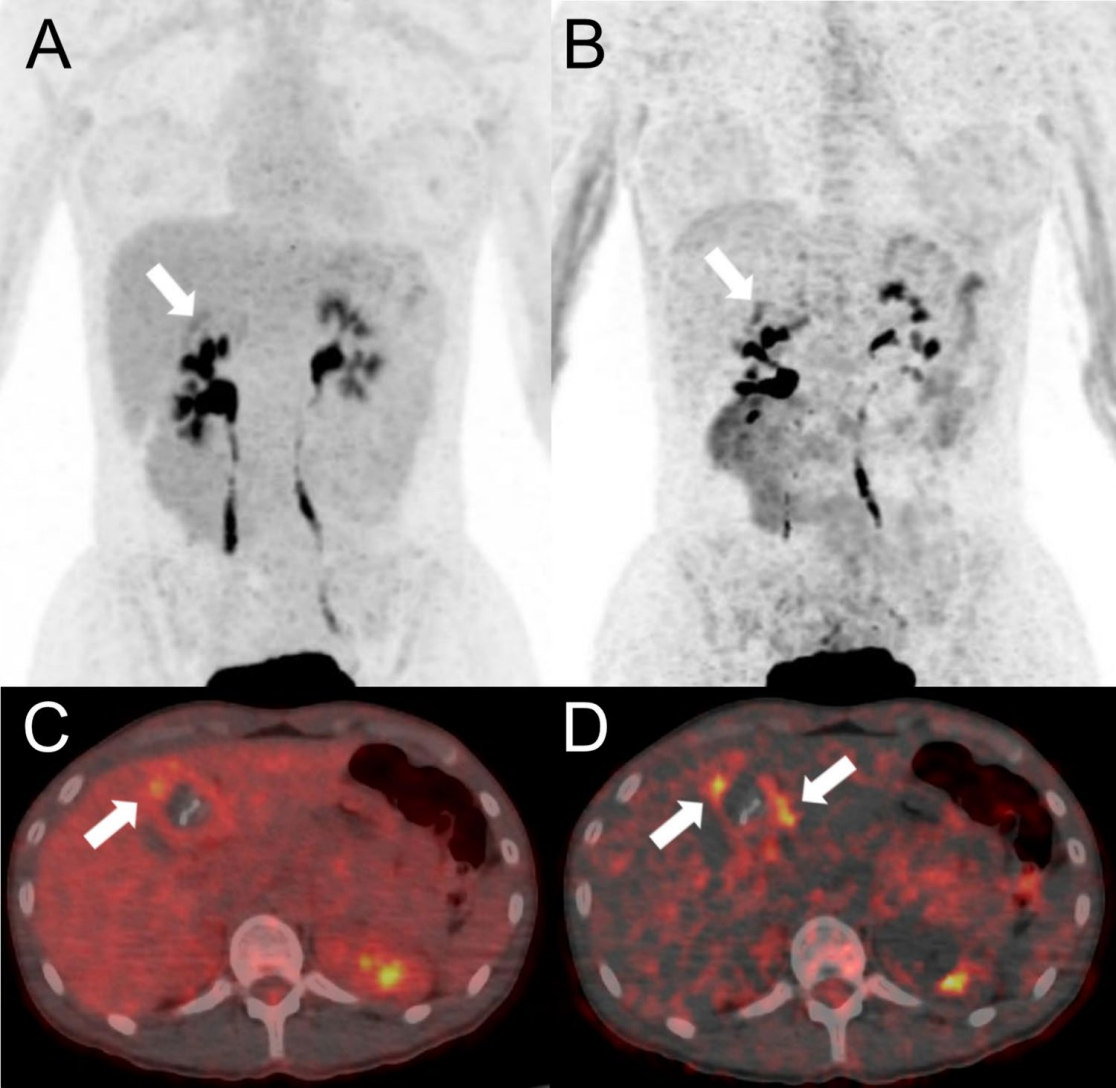
PET/CT performed for staging of AE in a 26-year old woman (patient 10 in Table 1) showed mild metabolic activity (in maximum intensity reconstructions of PET, **A** and **B**) and fused PET/CT images (**C** and **D**) in a partially calcified lesion in the left liver lobe. In standard static PET/CT images (A and C), SUVmax of the AE manifestation was 4.1, and the lesion-to-background ratio (SUVratio) was 1.3. For WBD PET/CT (**B** and **D**), the respective value in the AE manifestation was 15.7, and the lesion-to-background ratio was comparatively higher (Patlak ratio 2.6)

PET/CT was successfully performed with diagnostic image quality after intravenous injection of FDG in all patients (Figs. 1 and 2). The mean SUVmax in AE manifestations with the highest FDG uptake was 5.7 ± 3.1 (range: 3.2–13.9), compared to 3.2 ± 0.4 (range: 2.5–4.2) in non-infected liver tissue. Respective values for Patlak were 13.0 ± 8.6 (range: 2.7–35.5) and 4.9 ± 2.8 (range: 0.6–12.1) for MR_FDG_. The SUVratio (1.8 ± 1.1; range 1.0–5.2) was significantly lower (P < 0.001) than the MR_FDG_ ratio (3.2 ± 3.2; range 1.1–15.6). All individual values are presented in Table 1.

### Relation of quantitative PET/CT measurements and antibodies

The mean serum EgHF antibody units (AU) were 28.8 ± 24.3 (range 0.0–106), while the respective values for EgP were 29.8 ± 27.6 (range 0.0–103), and for Em-18 11.8 ± 26.1 (0.0–108); individual values are displayed in Table 1.

**Fig. 3:**
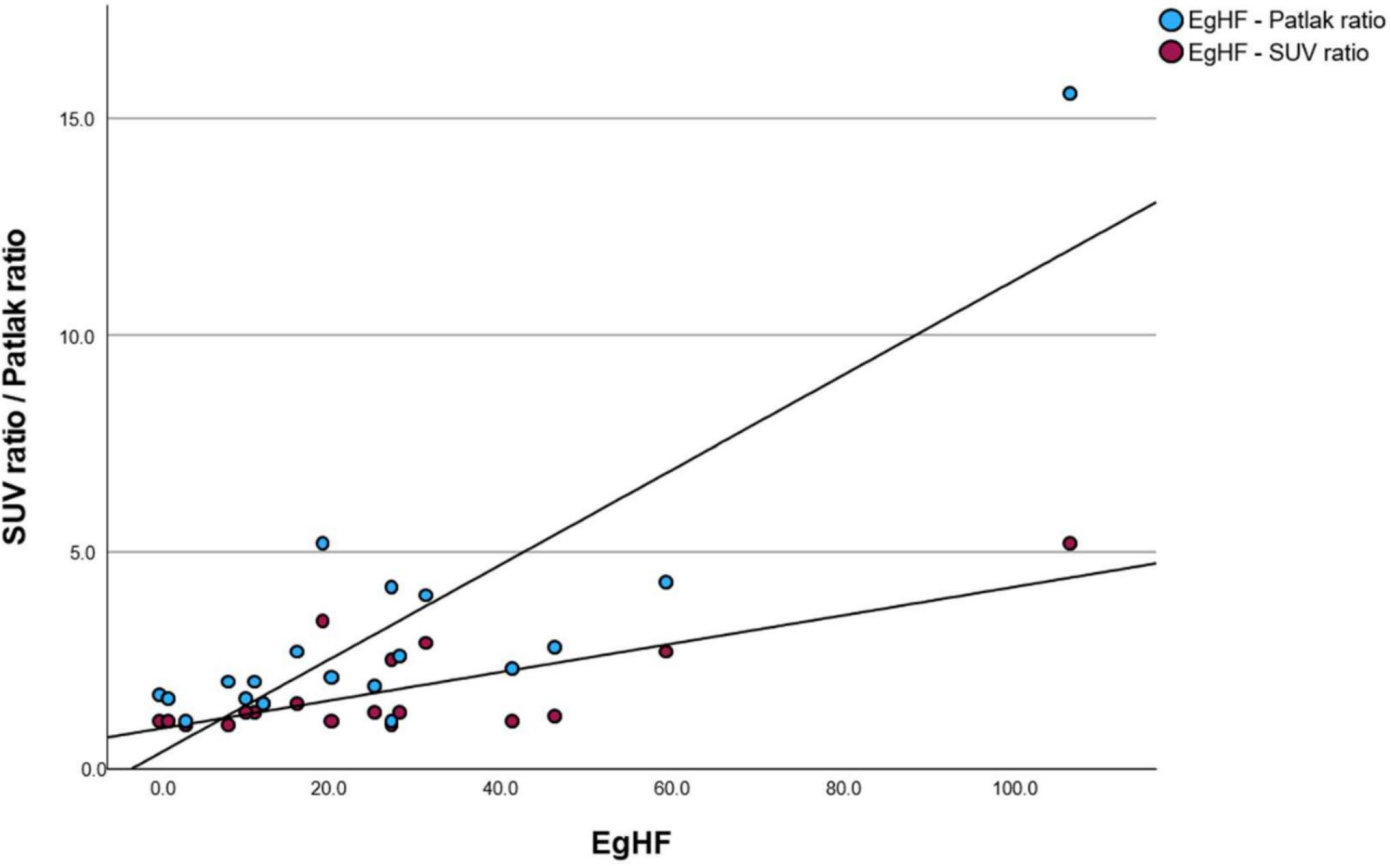
Scatter plot of EgHF AU levels against SUVratio and Patlak ratio, demonstrating significant correlation of metabolic activity of the AE manifestation measured in PET/CT with serum EgHF AU levels (SUVratio *r* = 0.76, *P* < 0.001; Patlak ratio *r* = 0.85, *P* < 0.001)

Ratios of metabolic activity of the AE manifestation measured in PET/CT were significantly correlated with serum EgHF AU levels (SUVratio *r* = 0.76, *P* < 0.001; Patlak ratio *r* = 0.85, *P* < 0.001; Fig. 3). Em18 significantly correlated with SUVratio (*r* = 0.51, *P* = 0.027); however, no correlation of Em-18 and Patlak ratio was found (*r* = 0.19, *P* = 0.42).

## Discussion

We analyzed the role of whole-body dynamic/Patlak parametric PET/CT imaging in patients with AE. Our study results are as follows: (i) WBD PET/CT images provide higher lesion-to-background contrast compared to standard static PET/CT images. (ii) The lesion-to-background contrast for both imaging techniques correlates significantly with serum EgHF antibodies.

Although PET/CT has been demonstrated as a reliable modality for staging, restaging, and therapeutic decision-making in patients with AE [22], serological markers (such as Em-18) are currently preferred over PET/CT in clinical practice. These markers typically offer comparable clinical information at lower cost and without radiation exposure. PET/CT is currently mainly utilized for two specific indications: (i) patients with active disease but negative Em-18 serology, and (ii) patients for whom long-term benzimidazole treatment is being considered for discontinuation.

Em-18 antibody is a highly specific marker for active infection and serves as the most important marker for diagnosis and therapy monitoring in AE [23, 24]. In a small subset of patients, Em-18 serology may occasionally be negative, despite the presence of active AE. In these cases, PET/CT is routinely employed for staging and therapy monitoring. Our results demonstrate that both imaging techniques demonstrate excellent correlation with EgHF antibodies, and that WBD PET/CT images provide higher lesion-to-background contrast compared to standard PET/CT images. Based on these findings, we hypothesize that the sensitivity and accuracy for staging and therapy monitoring may be further improved with this new technique. However, this hypothesis was beyond the scope of our current study and warrants further investigations.

In patients with inoperable AE, long-term benzimidazole treatment may be considered for discontinuation, as aborted forms of AE have been observed after years of therapy, suggesting that benzimidazole may be parasitocidal, and not just parasitostatic in some cases [6]. Initial attempts to discontinue benzimidazole therapy based on negative PET/CT follow-up scans, regardless of serology markers, were associated with a high rate of recurrence [12]. We may hypothesize that the potentially higher sensitivity of WBD PET/CT could reduce the recurrence rate in a similar study setting. In current clinical practice, the decision to discontinue benzimidazole therapy is usually based on a combination of negative PET/CT results and undetectable levels of Em-18 antibodies, a strategy that has been shown to be successful without recurrences in smaller study populations [25]. However, it is important to note that the introduction of WBD PET/CT may potentially jeopardize the success of benzimidazole therapy discontinuation. An increase in sensitivity with WBD PET/CT imaging may, in fact, represent a post hoc fallacy, leading to an unnecessary prolongation of benzimidazole therapy in some patients.

We performed both full dynamic acquisition with injection on the table followed by analysis with standard Patlak as well as truncated dynamic acquisitions resulting in a partial input function followed by analysis with relative Patlak. A full dynamic acquisition for 60 min, even though could provide superior quantification to standard SUV values, is a time-consuming alternative to standard static imaging or serological markers. Relative Patlak can provide indistinguishable lesion-to-background contrast compared to standard Patlak as the scaling in the MR_FDG_ resulting from the truncated input function cancels out in the ratio. Hence, relative Patlak analysis from a short dynamic acquisition could potentially substitute static acquisition metrics in the future, as the duration of such truncated dynamic scans could become similar to current static acquisitions when acquired on longer axial FOV scanner of increased sensitivity.

### Limitations of the study

Our present prospective study involved a relatively small number of patients, given the rarity of the disease. In addition, the study population was heterogeneous, including patients referred for initial staging and for following-up of AE, as well as a high number of patients with negative Em-18 marker, which limited the ability to draw conclusive statistical conclusions, as we detected only a weak correlation of Em-18 and SUVratio and no correlation between Em-18 and Patlak ratio. However, despite these limitations, we were able to statistically confirm the hypothesis that WBD PET/CT demonstrated higher lesion-to-background contrast in patients with AE.

## Conclusion

WBD PET/CT provides significantly higher lesion-to-background contrast compared to standard static PET/CT, and may, therefore, enhance sensitivity and accuracy in the assessment and reassessment of hepatic AE.

## Data Availability

All relevant data is given in Table 1.

## References

[1] Eckert J, Deplazes P. Biological, epidemiological, and clinical aspects of echinococcosis, a zoonosis of increasing concern. Clin Microbiol Rev. 2004;17:107–35. 14726458 10.1128/CMR.17.1.107-135.2004 PMC321468

[2] Brunetti E, Kern P, Vuitton DA, Writing Panel for the W-I. Expert consensus for the diagnosis and treatment of cystic and alveolar echinococcosis in humans. Acta Trop. 2010;114:1–16. 19931502 10.1016/j.actatropica.2009.11.001

[3] Hillenbrand A, Gruener B, Kratzer W, Kern P, Graeter T, Barth TF, et al. Impact of safe distance on long-term outcome after surgical therapy of alveolar echinococcosis. World J Surg. 2017;41:1012–8. 27822723 10.1007/s00268-016-3813-6

[4] Torgerson PR, Schweiger A, Deplazes P, Pohar M, Reichen J, Ammann RW, et al. Alveolar echinococcosis: from a deadly disease to a well-controlled infection. Relative survival and economic analysis in Switzerland over the last 35 years. J Hepatol. 2008;49:72–7. 18485517 10.1016/j.jhep.2008.03.023

[5] Ammann RW, Hirsbrunner R, Cotting J, Steiger U, Jacquier P, Eckert J. Recurrence rate after discontinuation of long-term mebendazole therapy in alveolar echinococcosis (preliminary results). Am J Trop Med Hyg. 1990;43:506–15. 2240375 10.4269/ajtmh.1990.43.506

[6] Wilson JF, Rausch RL, McMahon BJ, Schantz PM. Parasiticidal effect of chemotherapy in alveolar hydatid disease: review of experience with mebendazole and albendazole in Alaskan Eskimos. Clin Infect Dis. 1992;15:234–49. 1520758 10.1093/clinids/15.2.234

[7] Ammann RW, Fleiner-Hoffmann A, Grimm F, Eckert J. Long-term mebendazole therapy may be parasitocidal in alveolar echinococcosis. J Hepatol. 1998;29:994–8. 9875648 10.1016/s0168-8278(98)80129-3

[8] Ishizu H, Uchino J, Sato N, Aoki S, Suzuki K, Kuribayashi H. Effect of albendazole on recurrent and residual alveolar echinococcosis of the liver after surgery. Hepatology. 1997;25:528–31. 9049192 10.1002/hep.510250305

[9] Ammann RW, Ilitsch N, Marincek B, Freiburghaus AU, Swiss Echinococcosis Study Group. Effect of chemotherapy on the larval mass and the long-term course of alveolar echinococcosis. Hepatology. 1994;19:735–42. 8119701 10.1002/hep.1840190328

[10] Ammann RW, Renner EC, Gottstein B, Grimm F, Eckert J, Renner EL. Immunosurveillance of alveolar echinococcosis by specific humoral and cellular immune tests: long-term analysis of the Swiss chemotherapy trial (1976–2001). J Hepatol. 2004;41:551–9. 15532108 10.1016/j.jhep.2004.06.015

[11] Ammann RW, Stumpe KD, Grimm F, Deplazes P, Huber S, Bertogg K, et al. Outcome after discontinuing long-term benzimidazole treatment in 11 patients with non-resectable alveolar echinococcosis with negative FDG-PET/CT and anti-EmII/3-10 serology. PLoS Negl Trop Dis. 2015;9:e0003964. 26389799 10.1371/journal.pntd.0003964 PMC4577091

[12] Reuter S, Buck A, Manfras B, Kratzer W, Seitz HM, Darge K, et al. Structured treatment interruption in patients with alveolar echinococcosis. Hepatology. 2004;39:509–17. 14768005 10.1002/hep.20078

[13] Caoduro C, Porot C, Vuitton DA, Bresson-Hadni S, Grenouillet F, Richou C, et al. The role of delayed ^18^F-FDG PET imaging in the follow-up of patients with alveolar echinococcosis. J Nucl Med. 2013;54:358–63. 23303963 10.2967/jnumed.112.109942

[14] Patlak CS, Blasberg RG, Fenstermacher JD. Graphical evaluation of blood-to-brain transfer constants from multiple-time uptake data. J Cereb Blood Flow Metab. 1983;3:1–7. 6822610 10.1038/jcbfm.1983.1

[15] Karakatsanis NA, Lodge MA, Tahari AK, Zhou Y, Wahl RL, Rahmim A. Dynamic whole-body PET parametric imaging: I. concept, acquisition protocol optimization and clinical application. Phys Med Biol. 2013;58:7391–418. 24080962 10.1088/0031-9155/58/20/7391 PMC3941007

[16] Maurer A, Kotasidis F, Deibel A, Burger IA, Huellner MW. Whole-body ^18^ F-FDG PET/CT patlak parametric imaging of hepatic alveolar echinococcosis. Clin Nucl Med. 2023;48:1089–90. 37801583 10.1097/RLU.0000000000004878

[17] Ricard F, Cheson B, Barrington S, Trotman J, Schmid A, Brueggenwerth G, et al. Application of the Lugano classification for initial evaluation, staging, and response assessment of Hodgkin and non-Hodgkin lymphoma: the PRoLoG consensus initiative (part 1-clinical). J Nucl Med. 2023;64:102–8. 35835580 10.2967/jnumed.122.264106 PMC9841255

[18] Kern P, Wen H, Sato N, Vuitton DA, Gruener B, Shao Y, et al. WHO classification of alveolar echinococcosis: principles and application. Parasitol Int. 2006;55(Suppl):S283–7. 16343985 10.1016/j.parint.2005.11.041

[19] Kern P. Clinical features and treatment of alveolar echinococcosis. Curr Opin Infect Dis. 2010;23:505–12. 20683265 10.1097/QCO.0b013e32833d7516

[20] Junghanss T, da Silva AM, Horton J, Chiodini PL, Brunetti E. Clinical management of cystic echinococcosis: state of the art, problems, and perspectives. Am J Trop Med Hyg. 2008;79:301–11. 18784219

[21] Kronenberg PA, Deibel A, Gottstein B, Grimm F, Mullhaupt B, Meyer Zu Schwabedissen C, et al. Serological assays for alveolar and cystic echinococcosis-a comparative multi-test study in Switzerland and Kyrgyzstan. Pathogens. 2022. 10.3390/pathogens11050518. 35631039 10.3390/pathogens11050518 PMC9146094

[22] Husmann L, Gruenig H, Reiner CS, Deibel A, Ledergerber B, Liberini V, et al. Prediction of benzimidazole therapy duration with PET/CT in inoperable patients with alveolar echinococcosis. Sci Rep. 2022;12:11392. 35794149 10.1038/s41598-022-15641-5 PMC9259695

[23] Akira I. Serodiagnosis of alveolar echinococcosis: detection of antibody against EM18 in patients and rodents. Southeast Asian J Trop Med Public Health. 1997;28(Suppl 1):117–24. 9656361

[24] Tappe D, Sako Y, Itoh S, Frosch M, Gruner B, Kern P, et al. Immunoglobulin G subclass responses to recombinant Em18 in the follow-up of patients with alveolar echinococcosis in different clinical stages. Clin Vaccine Immunol. 2010;17:944–8. 20392888 10.1128/CVI.00026-10 PMC2884418

[25] Husmann L, Muehlematter UJ, Grimm F, Ledergerber B, Messerli M, Kudura K, et al. PET/CT helps to determine treatment duration in patients with resected as well as inoperable alveolar echinococcosis. Parasitol Int. 2021;83:102356. 33872794 10.1016/j.parint.2021.102356

